# Socio-economic disparities in clinical outcomes of transfusion-dependent β-thalassaemia patients

**DOI:** 10.1186/s41043-026-01291-0

**Published:** 2026-03-26

**Authors:** Demetris Avraam, Michael Angastiniotis, Elpidoforos S. Soteriades,, Androulla Eleftheriou

**Affiliations:** 1 Department of Public Health, University of Copenhagen, Copenhagen, Denmark; 2 Department of Public Health, Policy and Systems, University of Liverpool, Liverpool, UK; 3 Thalassaemia International Federation (TIF), Nicosia, Cyprus; 4 Healthcare Management Program, School of Economics and Management, Open University of Cyprus, 33 Giannou Kranidioti Ave, 2220 Latsia Nicosia, Cyprus

**Keywords:** β-thalassaemia, Socio-economic status, Health inequality, Clinical outcomes, Healthcare management, Thalassaemia international federation

## Abstract

**Background:**

Reducing inequalities among patients with inherited, chronic, and complex disorders requiring lifelong care remains a global public health priority. Despite medical advances, inequalities in healthcare access and quality persist for patients with β-thalassaemia. This study examined the associations between socio-economic indicators at the individual and country levels and clinical outcomes in β-thalassaemia patients using data from the International Thalassaemia Collaborative Assessment Patient Survey.

**Study design and methods:**

Data from 971 patients across 45 countries were analysed. Individual-level socio-economic status (SES) was assessed through patients’ educational attainment and employment status, while country-level SES was represented by the Human Development Index and the World Bank Income classification. Clinical outcomes included pre-transfusion haemoglobin levels, serum ferritin concentration, cardiac T2* score, and liver iron concentration. Inequalities were quantified using the Slope Index of Inequality, representing absolute differences in outcomes between patients of lowest and highest SES.

**Results:**

All SES indicators were positively associated with better clinical outcomes. Higher education and full-time employment corresponded to better levels of haemoglobin, ferritin, cardiac, and liver iron measures. Similarly, patients living in higher-income countries had better clinical measures compared to those living in lower-income countries. These findings indicate that socio-economic advantage is linked with improved disease management and monitoring quality.

**Discussion:**

Socio-economic determinants influence the clinical outcomes of transfusion-dependent β-thalassaemia patients, which in turn affect their social integration and quality of life. Reducing these disparities requires investment in equitable healthcare systems, multidisciplinary care, and disease-specific public health policies.

**Supplementary Information:**

The online version contains supplementary material available at 10.1186/s41043-026-01291-0.

## Introduction

Congenital disorders requiring lifelong treatment contribute significantly to the healthcare burden, particularly in regions where these conditions are highly prevalent. A notable example is β-thalassaemia, with an estimated one million patients worldwide; although in the absence of effective national registries, detailed epidemiological monitoring and prevention programs, this may be an underestimation [1]. Regions in which β-thalassaemia is endemic include the Mediterranean basin, the Middle East, and the whole of Asia [2, 3]. Although most patients live in low-resource countries, migration has increased the prevalence of β-thalassaemia in Western countries, where healthcare resources are adequate but experience with this rare inherited disease remains limited [4].

The need for complex medical monitoring and lifelong treatment to manage these patients on a regular basis has led to major discrepancies in medical and social outcomes, reflecting differences in healthcare system investment and capacity [5]. Without treatment, most severe forms of β-thalassaemia result in fatalities in early childhood, while with optimal modern medical care, patients can survive to advanced age with a good quality of life [6]. However, most of the patients worldwide do not receive optimal care. This is mainly due to blood shortages leading to inadequate transfusions and sub-optimal haemoglobin levels. Additional barriers include poor control of iron overload, often resulting from limited availability of chelating agents or ineffective chelation management, lack of adequate knowledge for medical management, failure to adopt international guidelines, and patient non-compliance with lifelong and cumbersome treatment protocols [7]. In addition to the above factors, there is often inadequate monitoring to detect and timely manage complications in vital organs mainly due to inadequate multidisciplinary care, lack of access to medical imaging and shortage of expert reference centres – an essential component of the effective management of such complex, chronic disorders [8, 9].

The consequences of sub-standard medical management include reduced quality of life and premature death. Such outcomes are evident by following various β-thalassaemia birth cohorts, since older patients, mainly those living in the Western world, have been treated in earlier life with older less effective regimens when knowledge and experience regarding treatment of this disorder were quite confined. However, even younger patients living in lower- and middle-income countries still follow such inadequate treatment protocols that are associated with higher rates of morbidity and premature mortality [6, 10–12].

While many studies have demonstrated the impact of social inequalities on people’s health and quality of life [13, 14], research specifically on β-thalassaemia patients is limited. In this study we aimed at examining the association between social factors at the individual level and economic indices at the country level in association with clinical outcomes of β-thalassaemia patients as indicators of healthcare quality. Particularly, we have examined the association between socio-economic status (education and employment) as well as aggregate measures at the country level such as the World Bank Income classification [15] and the Human Development Index introduced by the United Nations Development Programme [16], and their impact on clinical measures among β-thalassaemia patients across a large number of countries around the world.

### Study design and methods

Data used in the current study were retrieved from the cross-sectional analysis of the 2023 International Thalassaemia Collaborative Assessment (ITHACA) Patient Survey distributed via TIF networks. Details on inclusion/exclusion criteria and survey distribution methodology are described in a previous publication [17]. The information in the current study was retrieved from 45 countries in all six World Health Organization (WHO) regions. The survey was distributed among country members of the Thalassaemia International Federation (TIF) through their patient associations and healthcare professional networks. Participants were asked to complete an anonymous questionnaire without identifying personal information. The questionnaire included an introductory statement that the survey was anonymous and that the data would be kept confidential. It also stated that it was referring to thalassaemia patients over 15 years old, or the parents of patients with thalassaemia under the age of 15. In addition, it clarified that the survey was being conducted to support TIF’s mission to lobby for safe and better-quality treatment services for thalassaemia patients worldwide. The completion of the online questionnaire by each patient or their legal guardian was an expressed consent for participating in the anonymous survey. All research was performed in accordance with relevant guidelines/regulations and in accordance with the Declaration of Helsinki.

For the current analysis, we included patients aged 20 years or older who completed the TIF survey, reported receiving regular blood transfusions and had a diagnosis of either β-thalassaemia major or intermediate.

### Socio-economic indicators

Patients’ educational level and employment status were used as the measures of individual-level socio-economic status (SES) indicators. Educational attainment was reported via the question “*What is the highest level of school you have completed or the highest degree you have received?*” where participants selected one of several options. Details on the use of educational level as an SES indicator are presented in the supplementary materials (Table S3). Education was used as an indicator of socioeconomic position because it is a stable, widely recorded measure that strongly influences family resources and health behaviours, and it reflects quality of care in several ways. First, educational attainment is itself a marker of survival; in many populations, patients typically survive only into late adolescence or their early twenties, so having a cohort in which individuals complete tertiary education indicates substantially improved longevity. Second, higher educational attainment reflects a better quality of life, which is closely linked to effective clinical management. Third, it signals strong social integration, including social acceptance, family support, and psychosocial assistance from provided services. Effective patient adjustment and self-management depend on sustained education and support from an early age—outcomes that stem from a holistic approach to care. Employment status was reported via the question “*Which of the following categories best describes your employment status*” where the participants selected one of different options presented in the supplementary materials (Table S3).

At the country level, SES was represented by the World Bank Income (WBI) classification [15] and the United Nations Human Development Index (HDI) [16]. The WBI classifies countries into four income groups: low, lower-middle, upper-middle, and high-income. The HDI is a composite measure of average achievement in key dimensions of human development including: a long and healthy life, being knowledgeable and having a decent standard of living, and it classifies the countries to low, medium, high and very high levels. The HDI reflects a society’s development beyond economic growth alone. Some experienced countries possess adequate resources yet have not fully provided universal health coverage or ensured corresponding improvements in their population health. Infant mortality is another example, where high national income does not always translate into sufficiently low infant mortality rates. The HDI incorporates life expectancy, educational attainment, and a decent standard of living measured by gross national income per capita. It therefore captures both the quality of services available to the population and the broader level of socioeconomic development.

Each socio-economic indicator was converted to a scaled variable based on the cumulative distribution of participants across strata of the indicator, where each level was assigned a score between 0 and 1 representing the midpoint of its cumulative proportion. For example, if 20% of participants had low education, 30% medium, and 50% high, they would be allocated scores of 0.10 (0.20/2), 0.35 (0.20 + 0.30/2), and 0.75 (0.20 + 0.30 + 0.50/2), respectively.

### Clinical outcomes

Four indicators were used as clinical outcome measures for β-thalassaemia patients [18]: pre-transfusion haemoglobin (Hb) level, serum ferritin level, cardiac MRI T2* score, and liver iron concentration (LIC). Each variable was dichotomized as follows:

- Hb coded as 1 if the patient’s usual pre-transfusion Hb level was > 9 g/dL, and 0 otherwise.
- Ferritin coded as 1 if patient’s ferritin level was ≤ 1000 ng/mL, and 0 otherwise.
- Cardiac T2* coded as 1 if patient’s most recent T2* score was > 20 ms, and 0 otherwise.
- LIC coded as 1 if patient’s most recent LIC was < 7 mg Fe/g of dry weight, and 0 otherwise.

Inequalities were quantified using the Slope Index of Inequality (SII), which estimates the absolute difference in the prevalence of clinical outcomes between patients at the highest and lowest SES levels. The SII is calculated as the slope (gradient) of a linear regression describing the relationship between a ranked scaled socio-economic variable (from lowest to highest) and the prevalence of a health outcome, with a value of 0 representing no inequality. Weighted linear regression was also used as a sensitivity analysis with weights defined in each case by the number of subjects at each level of the socio-economic variables used.

## Results

**Fig. 1:**
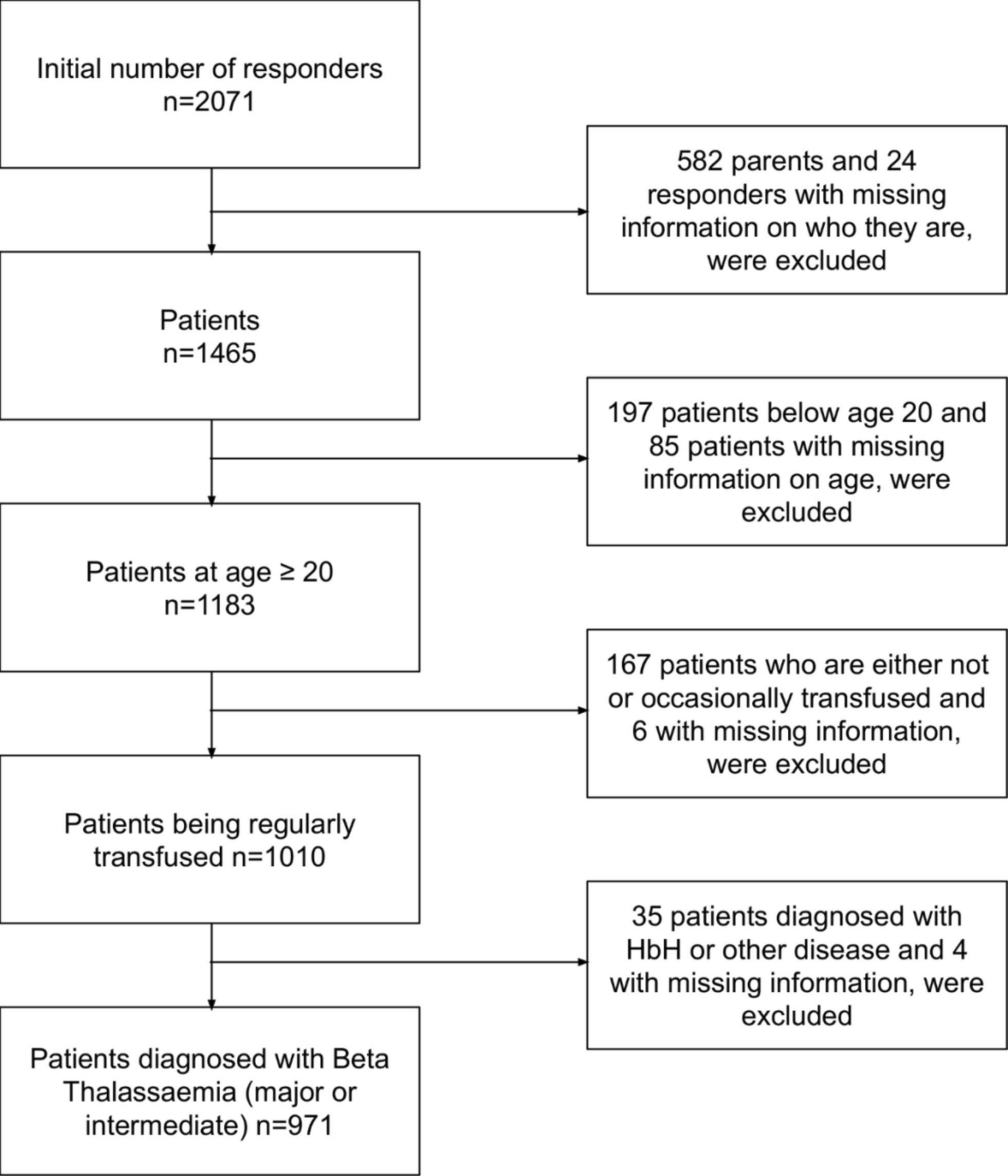
Flow chart of β-thalassaemia patients included in the current analysis

A total of 971 patients with β-thalassaemia were included in the analyses (Fig. 1, and Table S1 in Supplementary). The study sample consisted of 395 males and 567 females (9 patients had missing information about their gender). Overall, 18.7% of patients had a low educational level, 35.0% had a medium educational level, and 45.7% had a high educational level. Data on education were missing for five patients (0.5%). Employment was also limited among the study participants, with only 30.2% reporting full-time employment. The rest were either part-time employed (11.1%), unemployed (33.1%), or excluded from the analysis (25.1%) because they were retired (45 patients), disabled (104 patients), or not seeking work (95 patients). Five patients had missing information of employment (0.5%).

Based on the Human Development Index, 5.6%, 25.4%, 43.6% and 25.3% of participants were living in countries with low, medium, high, and very high development, respectively. According to the World Bank Income classification, 1.9%, 53.0%, 21.6% and 23.4% were living in low-, lower-middle-, upper-middle-, and high-income countries, respectively. Information of country was missing in one patient (0.1%).

**Fig. 2:**
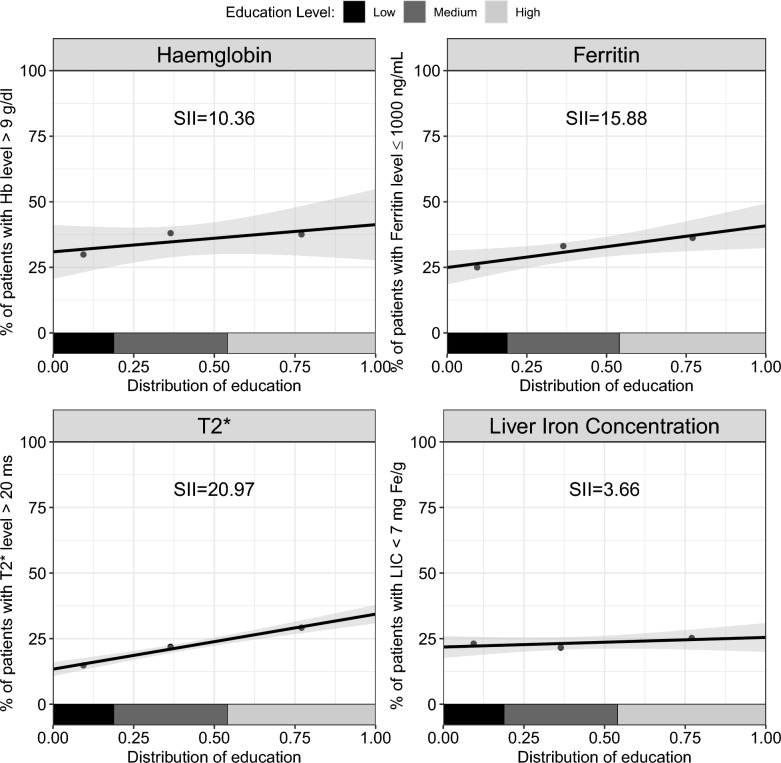
Socioeconomic gradient between patients’ educational level and the four clinical measures: haemoglobin (top left), ferritin (top right), cardiac MRI T2* score (bottom left), and liver iron concentration (bottom right)

As shown in Fig. 2, the prevalence of patients with pre-transfusion Hb level greater than 9 g/dL was 10.4% higher (Standard Error (SE) = 8.6) among those with high education compared to those with low education. Similarly, the prevalence of patients with serum ferritin level equal to or less than 1000 ng/mL was 15.9% higher (SE = 6.1), those with cardiac MRI T2* score greater than 20 ms was 21.0% higher (SE = 2.2), and those with LIC lower than 7 mg Fe/g was 3.7% higher (SE = 4.0) among the high-education group compared with the low-education group.

**Fig. 3:**
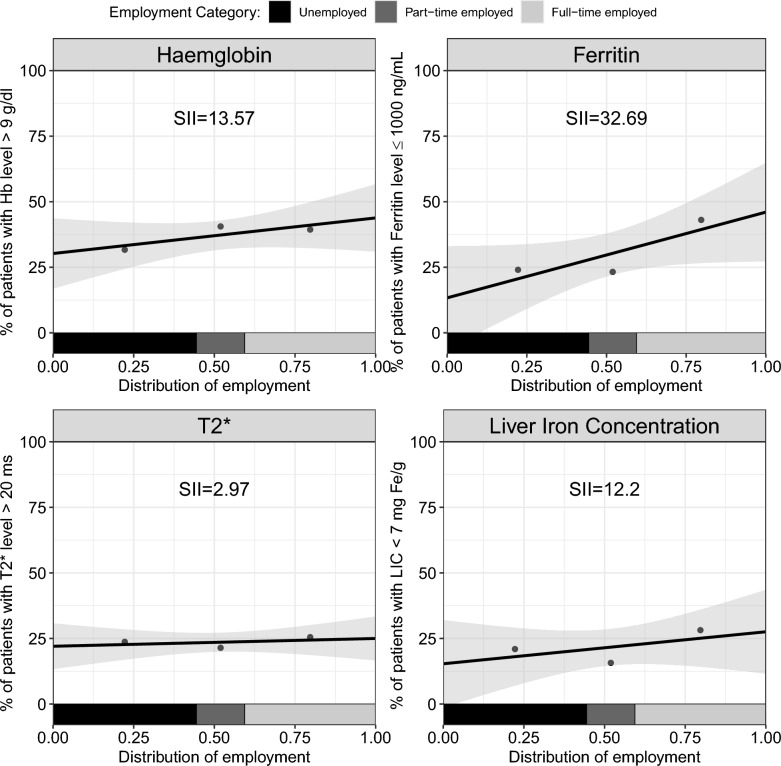
Socioeconomic gradient between patients’ employment status and the four clinical measures: haemoglobin (top left), ferritin (top right), cardiac MRI T2* (bottom left), and liver iron concentration (bottom right)

Figure 3 illustrates that patients employed full-time had a 13.6% higher prevalence (SE = 9.9) of pre-transfusion Hb level greater than 9 g/dL compared with unemployed patients. Similarly, the prevalence of patients with ferritin level equal to or less than 1000 ng/mL was 32.7% higher (SE = 21.4), those with cardiac MRI T2* score greater than 20 ms was 3.0% higher (SE = 6.4), and those with LIC lower than 7 mg Fe/g was 12.2% higher (SE = 18.1) among full-time employed compared to unemployed patients.

**Fig. 4:**
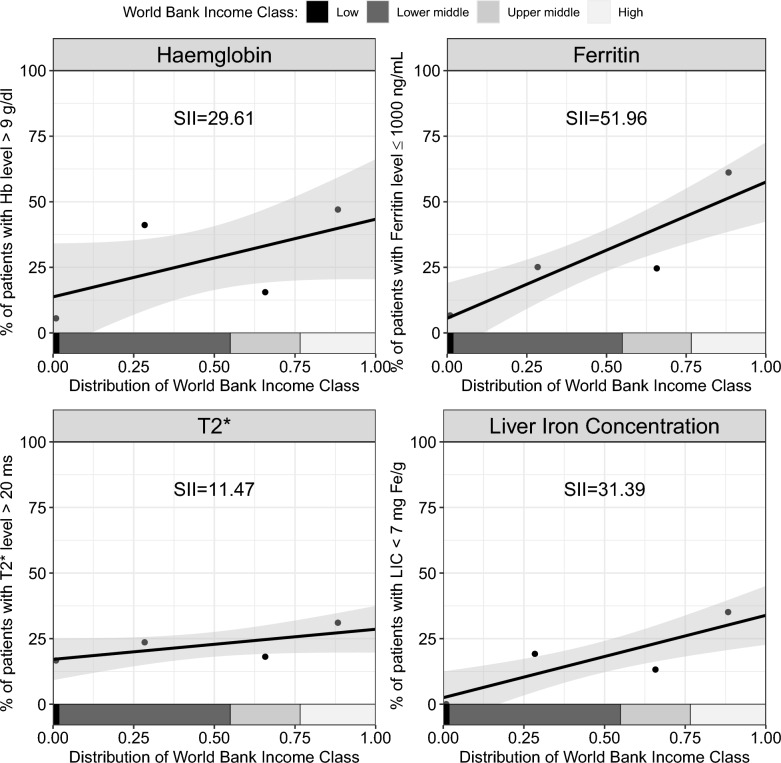
Socioeconomic gradient between counties’ World Bank Income (WBI) classification and the four clinical measures: haemoglobin (top left), ferritin (top right), cardiac MRI T2* score (bottom left), and liver iron concentration (bottom right)

As presented in Fig. 4, the prevalence of patients with pre-transfusion Hb level greater than 9 g/dL was 26.6% higher (SE = 29.7) among those living in high-income compared with low-income countries. Likewise, the prevalence of patients with ferritin level equal to or less than 1000 ng/mL was 52.0% higher (SE = 19.6), those with cardiac MRI T2* score greater than 20 ms was 11.5% higher (SE = 8.7), and those with LIC lower than 7 mg Fe/g was 31.4% higher (SE = 14.5) among patients living in high-income countries compared to those living in low-income countries based on WBI classification.

**Fig. 5:**
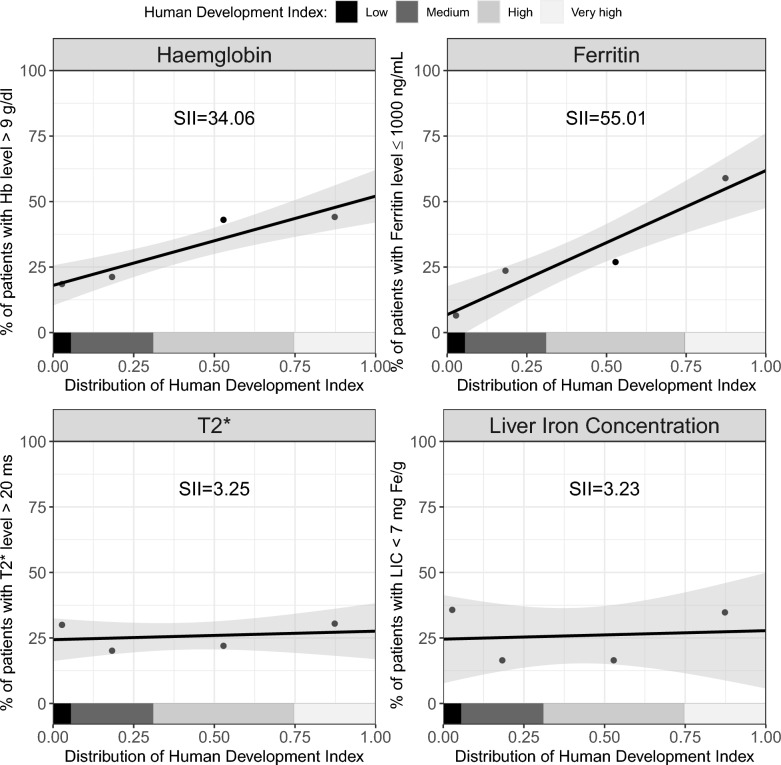
Socioeconomic gradient between counties’ Human Development Index (HDI) and the four clinical measures: haemoglobin (top left), ferritin (top right), cardiac MRI T2* score (bottom left), and liver iron concentration (bottom right)

In Fig. 5 we show that the prevalence of patients with pre-transfusion Hb level greater than 9 g/dL was 34.1% higher (SE = 9.2) among patients living in countries with very high HDI compared with those in low-HDI countries. Similarly, the prevalence of patients with ferritin level equal to or less than 1000 ng/mL was 55.0% higher (SE = 13.1), those with cardiac MRI T2* score greater than 20 ms was 3.3% higher (SE = 9.8), and those with LIC lower than 7 mg Fe/g was 3.2% higher (SE = 20.2) among patients living in countries with very high compared to those living in countries with low HDI.

The sensitivity analyses using a weighted linear regression showed similar inequalities (Figures S1-S4 in the Supplementary materials). While the magnitude of the SII slightly changed, the direction remained positive indicating that β-thalassaemia patients with advantaged socioeconomic position or living in advantaged countries had better clinical outcomes compared to patients with less advantaged conditions (Table S2 in the Supplementary materials).

## Discussion

Using four different socio-economic indicators to approximate the social ranking of β-thalassaemia patients across 46 countries in all WHO regions, this study identified a consistent positive gradient between both individual- and country-level socio-economic indicators and better clinical outcome measures. Specifically, higher educational attainment, full-time employment, residence in countries with stronger economies (higher WBI classification), and higher levels of human development index (HDI) were all associated with better clinical outcomes. Well-treated patients are those who comply with international guidelines regarding pre-transfusion haemoglobin levels, lower ferritin levels, lower cardiac iron concentration, and lower liver iron concentration. These parameters constitute widely accepted clinical indicators as described in the international guidelines edited by the Thalassaemia International Federation [19].

Both individual and aggregate socio-economic measures were positively correlated with better clinical outcomes among β-thalassaemia patients worldwide. This consistent pattern indicates that countries with stronger economies and well-resourced healthcare systems are more likely to sustain multidisciplinary medical services needed to achieve state-of-the art medical management of thalassaemia patients. Conversely, less developed countries with lower-resource settings face greater challenges in achieving recommended international treatment standards, resulting in poorer clinical outcomes [17]. Ineffective management leads to organ complications—particularly affecting the heart, liver, and endocrine glands—requiring additional interventions and long-term care [20]. These disparities are reflected in reduced survival, diminished quality of life, and ultimately greater societal and healthcare costs. At the same time, such medical neglect is likely to have an overall negative impact on countries’ economies since better-treated patients are more likely to become wage earners while inadequately treated patients will be more likely to remain unemployed and in need for financial support, disability pensions, and additional social services on top of lost earnings.

From our everyday interaction and experience—not directly drawn from this dataset—the issues faced in low socio-economic environments are numerous and interconnected. Haemoglobin disorders often receive low prioritization in policymaking because health authorities tend to focus on other pressing concerns such as infectious diseases and cardiovascular conditions. These disorders are also still widely viewed as incurable/fatal childhood illnesses, reflecting insufficient education and awareness among public health officials. Effective care requires substantial and reliable supplies of blood and medications yet achieving safe and adequate blood provision demands careful oversight of donation practices and rigorous quality control in blood banks, which do not always meet WHO or the International Society of Blood Transfusion (ISBT) standards. Systemic failures and underinvestment further undermine access to essential, often costly, chelating agents. Compounding these challenges is the limited social support available to patients, making it difficult for them to maintain adherence to the daily and lifelong treatments required for proper management of their condition.

Such considerations are constantly presented by the Thalassaemia International Federation so that more countries and more treatment centres can evaluate the cost of treatment with the purpose of assisting health authorities in correctly budgeting and adequately supporting the health and medical management of β-thalassaemia patient populations globally [21, 22]. Our findings suggest that prioritising management of complex chronic conditions such as β-thalassaemia, according to international standards, may be cost-effective in the long term by safeguarding the sustainability of health and social care systems [23].

Cost-effectiveness is expressed in terms of dollar cost per discounted healthy life years gained or the ratio of cost to health benefits or DALYs (Disability-adjusted life years) gained [24]. In this study we have used measures which do not refer to state evaluations or specific economic measures but simple indicators that patients themselves can understand and respond to. This may be regarded as a weakness of the study since patient impressions may not be accurate and measures that are related to clinical care such as cardiac MRI T2* and LIC were not available or known to the total patient population. Nevertheless, the clinical indicators are well recognised as prognostic of good or poor outcomes in β-thalassaemia. Nevertheless, iron overload and effective chelation is the main example [25–27]; pre-transfusion haemoglobin likewise is a predictor of complications [28]. The role of MRI measurements in regulating iron in vital organs has also been well documented [29]. Another limitation of our study might be the introduction of selection bias as the response rate might have been higher within the group of more educated patients. However, this does not affect our conclusions since inequalities based on country-level income and human development indices where similar to the inequalities seen based on individual socioeconomic indicators.

Based on the study findings, the Thalassaemia International Federation is able to advocate in a more focused and tailored way to the patients’ perceived needs to countries and social authorities around the world, urging them to develop new and/or strengthen existing disease-specific multidisciplinary programmes of comprehensive patient management. In addition, our findings support the notion that the implementation of related public health polices including blood transfusion services require a more targeted health/social budgeting to support β-thalassaemia patient services in national health agendas. The ultimate aim is to reduce inequality which is evident not only from the findings of the current study but also from the age distribution of patients [30] mainly occurring in low-middle-income countries. To date, the need of β-thalassaemia patients for equal access to quality, safe, effective and appropriate health and social care are significantly neglected.

### Implications for practice

Our study provides valuable mapping of the impact of social inequalities on measurable clinical outcomes of β-thalassaemia patients, highlighting the urgency of policy makers to transform research findings into small but feasible implementation steps to reduce such negative SES gaps. For example, standardized transfusion programs with technology assisted scheduling / recall may reduce discrepancies on Hb transfusion levels towards achieving the targeted outcome for all (Hb > 9 g/dL). Brief chelation-adherence support interventions, along with streamlined MRI access pathways, and modest financial / transport assistance for low-SES β-thalassaemia patients, could significantly narrow existing disease-management gaps and reach better clinical outcomes with respect to standard treatment goals (ferritin ≤ 1000 ng/mL, T2* > 20 ms, LIC < 7 mg/g). Often, distance to healthcare centers, overly complicated referral processes, and indirect costs not covered by health insurance may pose significant barriers to effective care. Additional social equity-stratified metrics such as monitoring of educational attainment and creating special employment pathways for such patients (e.g., aiming to narrow education/employment gradients over a period of 6–12 months), could definitely improve patients’ overall health, including lower disease-specific complications and higher longevity with better quality of life.

To this point, we may add the need for community involvement and advocacy since the clinical and social implications of such lifelong conditions may not be clearly understood by policy makers and health administrators. Understanding the true burden of disease in all its aspects (personal, clinical, psychological, and social), through epidemiological studies and surveillance outputs, could encourage and facilitate the collaboration of health professionals, policy makers and patient-support organizations. Finally, effective services should be implemented through appropriate health technology assessment mechanisms, optimal resource utilization (prevention, use of generic medications, more use of curative approaches etc.) and careful consideration of innovative therapies.

## Conclusions

This study provides international evidence supporting that individual SES and country-level economic development indices are strongly associated with clinical outcomes in β-thalassaemia patients. Strengthening healthcare infrastructure, providing multidisciplinary support, and implementing disease-specific social policies could substantially improve patients’ clinical outcomes. Health authorities and governments, in collaboration with WHO and regional bodies, should prioritize equitable access to high-quality healthcare services for all patients with β-thalassaemia. At the same time, countries are encouraged to honour their commitments to achieve United Nations Sustainable Development Goals 2030 aiming to achieve a better world for every human being irrespective of their age, sex, culture, colour, language, religion and health status.

## Supplementary Information

Additional file 1.

## Data Availability

Data are available from the corresponding author upon request.
