# Supplementary material for: Socio-economic disparities in clinical outcomes of transfusion-dependent β-thalassaemia patients
Source: J Health Popul Nutr. 2026 Mar 26;45:126. doi: 10.1186/s41043-026-01291-0 (PMC13147558; doi:10.1186/s41043-026-01291-0)
Supplement: Supplementary file 1 — Additional file 1. [file 41043_2026_1291_MOESM1_ESM.docx]

**Supplementary Materials**

**Table S1:** Country-specific characteristics of the total sample size of 971 patients used in the analysis. Country information was missing for one patient.

| **Country** | **Sample Size** | **WHO region** | **WBI** | **HDI** |
| --- | --- | --- | --- | --- |
| Albania | 8 | European Region | Upper middle income | High |
| Algeria | 5 | African Region | Lower middle income | High |
| Australia | 1 | Western Pacific Region | High income | Very high |
| Azerbaijan | 4 | European Region | Upper middle income | High |
| Bangladesh | 17 | South-East Asian Region | Lower middle income | Medium |
| Belgium | 1 | European Region | High income | Very high |
| Bulgaria | 1 | European Region | Upper middle income | High |
| China | 1 | Western Pacific Region | Upper middle income | High |
| Cyprus | 14 | European Region | High income | Very high |
| Denmark | 2 | European Region | High income | Very high |
| Egypt | 58 | Eastern Mediterranean Region | Lower middle income | High |
| France | 1 | European Region | High income | Very high |
| Germany | 16 | European Region | High income | Very high |
| Greece | 33 | European Region | High income | Very high |
| India | 81 | South-East Asian Region | Lower middle income | Medium |
| Indonesia | 33 | Western Pacific Region | Upper middle income | High |
| Iran | 224 | Eastern Mediterranean Region | Lower middle income | High |
| Iraq | 116 | Eastern Mediterranean Region | Upper middle income | Medium |
| Italy | 50 | European Region | High income | Very high |
| Jordan | 22 | Eastern Mediterranean Region | Lower middle income | High |
| Kuwait | 6 | Eastern Mediterranean Region | High income | Very high |
| Lebanon | 8 | Eastern Mediterranean Region | Lower middle income | High |
| Malaysia | 8 | Western Pacific Region | Upper middle income | Very high |
| Maldives | 27 | South-East Asian Region | Upper middle income | High |
| Mauritus | 1 | African Region | Upper middle income | Very high |
| Morocco | 2 | Eastern Mediterranean Region | Lower middle income | Medium |
| Nepal | 12 | South-East Asian Region | Lower middle income | Medium |
| North Macedonia | 1 | European Region | Upper middle income | High |
| Pakistan | 52 | Eastern Mediterranean Region | Lower middle income | Low |
| Palestine | 18 | Eastern Mediterranean Region | Lower middle income | High |
| Philippines | 3 | Western Pacific Region | Lower middle income | Medium |
| Romania | 20 | European Region | High income | Very high |
| Saudi Arabia | 45 | Eastern Mediterranean Region | High income | Very high |
| Singapore | 1 | Western Pacific Region | High income | Very high |
| Spain | 8 | European Region | High income | Very high |
| Sri Lanka | 6 | South-East Asian Region | Lower middle income | High |
| Switzerland | 1 | European Region | High income | Very high |
| Syria | 16 | Eastern Mediterranean Region | Low income | Medium |
| Trinidad and Tobago | 4 | Region of the Americas | High income | Very high |
| Tunisia | 7 | Eastern Mediterranean Region | Lower middle income | High |
| Turkey | 10 | European Region | Upper middle income | Very high |
| United Arab Emirates | 21 | Eastern Mediterranean Region | High income | Very high |
| United Kingdom | 1 | European Region | High income | Very high |
| United States | 2 | Region of the Americas | High income | Very high |
| Yemen | 2 | Eastern Mediterranean Region | Low income | Low |

**Sensitivity Analysis:** Calculating the Slope Index of Inequality using weighted linear regressions

**Figure S1:** Socioeconomic gradient between patients’ educational level and the four clinical measures: haemoglobin (top left), ferritin (top right), cardiac MRI T2* score (bottom left), and liver iron concentration (bottom right). The line fitting the data is derived by weighted linear regression.

 **Figure S2:** Socioeconomic gradient between patients’ employment status and the four clinical measures: haemoglobin (top left), ferritin (top right), cardiac MRI T2* (bottom left), and liver iron concentration (bottom right). The line fitting the data is derived by weighted linear regression.

**Figure S3:** Socioeconomic gradient between counties’ World Bank Income (WBI) classification and the four clinical measures: haemoglobin (top left), ferritin (top right), cardiac MRI T2* score (bottom left), and liver iron concentration (bottom right). The line fitting the data is derived by weighted linear regression.

**Figure S4:** Socioeconomic gradient between counties’ Human Development Index (HDI) and the four clinical measures: haemoglobin (top left), ferritin (top right), cardiac MRI T2* score (bottom left), and liver iron concentration (bottom right). The line fitting the data is derived by weighted linear regression.

**Table S2:** Comparison between the estimate of the Slope Index of Inequality (SII) and its standard error (SE) as derived by the linear regression in the main analysis (Figures 2-5) and by the weighted linear regression in the sensitivity analysis (Figures S1-S4).

|  |  | **Main analysis** | | **Sensitivity analysis** | |
| --- | --- | --- | --- | --- | --- |
| **Socioeconomic factor** | **Clinical outcome** | **SII** | **SE** | **SII** | **SE** |
| Education | Haemoglobin | 10.36 | 8.57 | 7.90 | 8.41 |
|  | Ferrtin | 15.88 | 6.12 | 13.77 | 5.96 |
|  | Cardiac MRI T2* | 20.97 | 2.23 | 20.22 | 2.18 |
|  | Liver iron concentration | 3.66 | 3.98 | 5.46 | 3.81 |
| Employment | Haemoglobin | 13.57 | 9.86 | 13.54 | 6.66 |
|  | Ferrtin | 32.69 | 21.35 | 32.97 | 14.22 |
|  | Cardiac MRI T2* | 2.97 | 6.43 | 3.08 | 4.65 |
|  | Liver iron concentration | 12.20 | 18.07 | 13.19 | 13.07 |
| World Bank Income Class | Haemoglobin | 26.61 | 29.66 | -0.31 | 33.19 |
|  | Ferrtin | 51.96 | 19.58 | 48.99 | 24.28 |
|  | Cardiac MRI T2* | 11.47 | 8.69 | 8.75 | 9.98 |
|  | Liver iron concentration | 31.39 | 14.53 | 23.20 | 13.62 |
| Human Development Index | Haemoglobin | 34.06 | 9.19 | 34.16 | 13.01 |
|  | Ferrtin | 55.01 | 13.07 | 51.73 | 18.17 |
|  | Cardiac MRI T2* | 3.25 | 9.78 | 9.74 | 8.76 |
|  | Liver iron concentration | 3.23 | 20.22 | 22.39 | 17.38 |

**Table S3:** Description of the details related to the educational level and employment status used as socio-economic status indicators in the study.

| Educational level | Educational attainment was reported via the question “What is the highest level of school you have completed or the highest degree you have received?” where participants selected one of the following options: (a) less than high school degree, (b) high school degree or equivalent (e.g., GED), (c) some college but no degree, (d) bachelor degree, (e) master degree, (f) doctoral degree, and (g) vocational school. For the analysis, education was categorized into three levels: ‘Low’ if they selected option (a), ‘Medium’ if they selected one of the options (b), (c) or (g), and ‘High’ if they selected one of the options (d), (e) or (f). |
| --- | --- |
| Employment Status | Employment status was reported via the question “*Which of the following categories best describes your employment status*” where the participants selected one of the following six options: (a) employed, working full-time, (b) employed, working part-time, (c) not employed, looking for work, (d) not employed, not looking for work, (e) retired, and (f) disabled, not able to work. For statistical analyses, employment status was categorized into three levels: ‘Unemployed’ if they selected option (c), ‘Part-time employed’ if they selected option (b) and ‘Full-time employed’ if the selected option (a). Participants who selected options (d), (e) and (f) were excluded from the analysis involving employment to ensure an indicator with hierarchical order from lower to higher levels of employment status. |

:.
